# Common symptoms in pregnancy and their correlations with anemia

**DOI:** 10.3389/fgwh.2026.1554655

**Published:** 2026-10-26

**Authors:** Yi Cheng Hou, Lu Lu Zhao, Ying Guang Zhang, Jing Hui Wu, Chi Hui Bai

**Affiliations:** 1 Department of Nutrition, Taipei Tzu Chi Hospital, Buddhist Tzu Chi Medical Foundation, New Taipei City, Taiwan; 2 Department of Pediatrics, Taipei Tzu-Chi Hospital, Buddhist Tzu-Chi Medical Foundation, New Taipei City, Taiwan; 3 Department of Obstetrics and Gynecology, Taipei Tzu-Chi Hospital, Buddhist Tzu-Chi Medical Foundation, New Taipei City, Taiwan; 4 Department of Public Health, College of Medicine, Taipei Medical University, Taipei, Taiwan

**Keywords:** anemia, dietary iron, hemoglobin, pregnancy, symptoms

## Abstract

**Introduction:**

Anemia is a common health problem that affects all age groups, yet it is particularly prevalent in women of reproductive age. Some symptoms of anemia may be misinterpreted as symptoms related to pregnancy. We aim to explore the association between common symptoms present during pregnancy and clinical anemia.

**Methods:**

This cross-sectional study analyzed data from 187 women who were recruited during their pregnancy.

**Results:**

A higher percentage of those who reported symptoms of anemia, such as tiredness and dizziness, were identified to have clinical anemia (20.7% in mildly anemic individuals and 10.0% in moderately anemic individuals, *p* *=* 0.023). Bloating (31.0% in mildly anemic individuals, 70.0% in moderately anemic individuals) and heartburn (17.0% in mildly anemic individuals) were also more common in mothers who experienced anemia during pregnancy. However, these differences did not reach statistical significance (*p* *=* 0.65 and *p* *=* 0.052, respectively).

**Conclusion:**

Self-perceived anemia symptoms were reported more frequently among pregnant individuals with clinical anemia. These findings suggest that when anemia symptoms are present, anemia screening should be performed whenever possible to confirm the presence of clinical anemia. This may facilitate early detection and intervention to mitigate the negative effects of anemia on the mother.

## 1 Introduction

While the number of cases has been decreasing, anemia remains a significant global health problem that affects people of all ages and sexes. A study conducted in 2021 discovered that poor nutrition is the leading cause of anemia in most regions (1). It is worth noting that East Asia has one of the highest ratios of years lived with disability due to anemia burden for female individuals compared to male individuals, demonstrating the impact of anemia on health challenges (1). Anemia can have various causes; however, deficiencies in micronutrients, such as iron, folate, and vitamin B12, are well-known culprits. Anemia resulting from parasitic infections is also common in developing countries (2). Nevertheless, iron deficiency anemia remains the most serious and widespread deficiency illness globally (3).

According to the most recent Nutrition and Health Survey in Taiwan (NAHSIT), the prevalence of anemia in Taiwanese women over 13 is approximately 4.0%, peaking at 14.0% in women of childbearing age (19–44 years old) (4). This prevalence gradually decreases as women age. The higher prevalence of anemia and iron-deficient anemia in women of childbearing age is likely due to blood loss during menstruation, a phenomenon observed worldwide. In lower-income countries, the prevalence of anemia in women of childbearing age is even more alarming, affecting a staggering 60% of this population (5, 6). Anemia during pregnancy is associated with numerous discomforts and adverse effects on the mother, which will be further discussed.

Dietary iron intake is a modifiable factor that, when addressed, can significantly improve public health. The importance of adequate dietary iron intake and anemia prevention has been extensively documented and proven. A recent review by O'Toole et al. revealed that despite the high prevalence of anemia, only about half of the international guidelines include recommendations related to iron intake (7). Referring specifically to the recommended dietary intakes for the Taiwanese population, the recommended iron intake during the third trimester and lactation is advised to increase by 200%, totaling 45 mg of dietary iron per day (8).

Multiple studies have emphasized the significance of starting pregnancy with sufficient iron stores to prevent the adverse effects of anemia, rather than relying solely on supplementation to treat anemia during pregnancy (9). In fact, iron supplementation during pregnancy may not be sufficient if mothers begin pregnancy with inadequate iron stores. Given the urgency of addressing this condition in the population, exploring the relationship between anemia and pregnancy symptoms is crucial. This enables pregnant individuals to be more aware of their dietary intake and any anemia-related symptoms that may go unnoticed. It also facilitates informed decision-making by governments and healthcare professionals regarding pregnancy care.

## 2 Materials and methods

### 2.1 Study participant recruitment and data collection

This cross-sectional study enrolled pregnant women from the Outpatient Department of Obstetrics and Gynecology, Taipei Tzu-Chi Hospital, New Taipei City, Taiwan (R.O.C.), between November 2018 and August 2019. The participants were recruited through convenience sampling. The inclusion criteria were as follows: (1) Pregnant women aged 15–45 with no major comorbidities (e.g., cancer, diabetes, cardiovascular disease); (2) complete anthropometric and biochemical data. All participants provided written informed consent on the day of recruitment. A total of 191 pregnant women were recruited for this study through convenience sampling.

On the day of recruitment, participants completed questionnaire which was identical to the one used in the Pregnant Women Nutrition Status Tracking Program, a subsection of the NAHSIT, initiated by the Taiwan Ministry of Health and Welfare from 2017 to 2019 (4). The questionnaire includes questions regarding general socioeconomic status and closed-ended questions regarding pregnancy-related symptoms. Biochemical values of mothers were obtained on the day of subject recruitment via a blood test as part of the routine checkup procedure during pregnancy. Outcome post-pregnancy was obtained from medical records during outpatient follow-up until childbirth. After analyzing the completeness of the information, 187 participants were eligible for inclusion in this study.

The diagnosis of iron-deficiency anemia and the severity of anemia were grouped according to the serum hemoglobin (Hb) concentration using the criteria defined by the World Health Organization in the Vitamin and Mineral Nutrition Information System (10). A plasma Hb level of more than 10.9 g/dL during any pregnancy trimester was classified as adequate; 10.0 g/dL ≤Hb ≤10.9 g/dL was classified as mild anemia; 7.0 g/dL ≤Hb ≤9.9 g/dL was classified as moderate anemia; Hb <7.0 g/dL was classified as severe anemia. According to this classification, no individual was identified as having severe anemia. Body mass index (BMI) grouping was performed according to the adult BMI criteria defined by Taiwan's Ministry of Health and Welfare as follows: (1) BMI <18.5 kg/m^2^ as underweight; (2) 18.5 kg/m^2^ ≤BMI <24 kg/m^2^ as normal body weight; (3) 24 kg/m^2^ ≤BMI <27 kg/m^2^ as overweight; (4) BMI ≥27 kg/m^2^ as obese.

### 2.2 Research ethics review

The study protocol was approved by the institutional review board (Approval Code: 07-XD-096), and funding was provided under grant number TCRD-TPE-112-RT-9.

### 2.3 Data analysis

Data analyses were conducted using R Foundation for Statistical Computing software, version 4.1.1. According to the variance and distribution of the data, the appropriate statistical analysis method was applied, including the Student's *t*-test, Wilcoxon rank-sum test, or chi-square test. The correlation coefficient was calculated using Pearson's or Kendall's rank correlation tests whenever appropriate. Results of categorical variables were presented as *n (%)*, with *n* representing the number of people in a certain group. The results of continuous variables were presented as mean ± SD. Correlation strength was classified as follows: 0.2–0.39 as weak, 0.40–0.59 as moderate, 0.6–0.79 as strong, and 0.8–1 as very strong. Subgrouping according to different criteria (including pregnancy trimester, formal education, and income, among others) was done to control confounders. Missing data were removed from the analysis. All statistical tests considered a *p*-value of *<*0.05 as denoting statistical significance, with a 95% confidence interval. The STROBE checklist has been utilized in this study to ensure comprehensive reporting.

## 3 Results

A total of 191 pregnant women were recruited in this study. After excluding participants with incomplete data, 187 pregnant women were included in the study, with 39 (20.9%) classified as anemic and 148 defined as non-anemic based on serum Hb levels. The baseline data of the participants collected on the day of recruitment are shown in Table 1. Data for parity (1 participant), education (2 participants), income (3 participants), and iron supplementation (12 participants) were excluded due to incomplete responses in the corresponding questionnaire sections.

**Table 1:** Baseline data of participants.

Anemia present				
Variable	All patients (*n* = 187)	Yes (*n* = 39)	No (*n* = 148)	*p*-value
Age	33.4 ± 4.2	33.3 ± 4.7	33.4 ± 4.1	0.95^a^
Hb (g/dL)	11.84 ± 1.2	10.2 ± 0.7	12.3 ± 0.8	<0.001^b^^,^*
Pre-pregnancy BMI	21.8 ± 3.5	22.2 ± 3.2	21.7 ± 3.5	0.24^b^
Underweight	23 (12.3)	3 (7.7)	20 (13.5)	0.74^c^
Normal	127 (67.9)	28 (71.8)	99 (66.9)	
Overweight	25 (13.4)	6 (15.4)	19 (12.8)	
Obese	12 (6.4)	2 (5.1)	10 (6.8)	
Parity^d^	1.5 ± 0.7	1.5 ± 0.7	1.6 ± 0.7	0.17
Trimester				
1	68 (36.4)	10 (25.6)	58 (39.2)	0.042^c^^,^*
2	67 (35.8)	12 (30.8)	55 (37.2)	
3	52 (27.8)	17 (43.6)	35 (23.6)	
Education^d^				
Primary school	2 (1.1)	1 (2.6)	1 (0.7)	0.29^c^
High school	8 (4.3)	0 (0.0)	8 (5.4)	
University	133 (71.5)	27 (69.2)	106 (72.1)	
Masters and above	43 (23.1)	11 (28.2)	32 (21.8)	
Income^d^				
Low (≤30,000 NTD)	10 (5.4)	2 (5.1)	8 (5.5)	0.76^c^
Low-mid range^d^ (30,000–59,999 NTD)	65 (35.1)	11 (28.2)	54 (37.0)	
Midrange^d^ (60,000–99,999 NTD)	74 (40.0)	17 (43.6)	57 (39.0)	
High (≥100,000 NTD)	36 (19.5)	9 (23.1)	27 (18.5)	
Baby's Sex^d^				
Male	50 (45.0)	6 (26.1)	44 (50.0)	0.041^a^^,^*
Female	61 (55.0)	17 (73.9)	44 (50.0)	
Iron Supplementation				
None	163 (93.1)	34 (91.9)	129 (93.5)	0.89^c^
Less than once a week	5 (2.9)	1 (2.7)	4 (2.9)	
Every day	7 (4.0)	2 (5.4)	5 (3.6)	

Hb, hemoglobin; NTD, new Taiwan Dollar; values presented as mean ± SD or *n* (%).

* *p*-value < 0.05.

^a^ Student's *t*-test.

^b^ Wilcoxon rank-sum test.

^c^ Chi-squared test.

^d^ Some data removed due to unavailability.

When stratified according to the anemia cut-off, the group with anemia had a lower mean Hb concentration of 10.2 ± 0.7 g/dL, compared to 12.3 ± 0.8 g/dL in those without clinical anemia (*p* *<* 0.001). The distribution of individuals in the anemic group in the first, second, and third trimesters was significantly different compared to those without anemia (*p* *=* 0.042). Specifically, among those with anemia, 43.6% were in the third trimester, while the first and second trimesters made up 25.6% and 30.8% of the anemic group, respectively. The proportion of fetal sex was different between the two groups, where 73.9% of anemic participants bore a female child, compared to 50.0% in the non-anemic group (*p* *=* 0.041). Other variables, including age, BMI, parity, education, income, and iron supplementation, did not differ significantly between the groups.

Next, we analyzed and compared the hematological parameters and nutritional markers correlated with anemia in the two groups. Table 2 presents the comparison of hematological markers and other anemia-related markers in the anemic and non-anemic groups. Most anemia biomarkers were significantly lower in the anemic group, including ferritin (24.2 ± 33.3 vs. 30.0 ± 26.2; *p* *=* 0.0021), red blood cell (RBC) count (3.96 ± 0.56 vs. 4.16 ± 0.43; *p* *=* 0.0048), hematocrit (HCT; 32.5 ± 2.29 vs. 37.3 ± 2.38; *p* *<* 0.001), mean corpuscular volume (MCV; 83.2 ± 9.4 vs. 90.0 ± 6.3; *p* *<* 0.001), mean corpuscular hemoglobin (MCH; 26.3 ± 3.6 vs. 29.6 ± 2.4; *p* *<* 0.001), mean corpuscular hemoglobin concentration (MCHC; 31.5 ± 1.2 vs. 32.9 ± 0.9; *p* *<* 0.001), and plasma folate (11.7 ± 6.6 vs. 14.9 ± 6.5; *p* *=* 0.0058). In contrast, total iron binding capacity (TIBC; 487 ± 119 vs. 438 ± 90; *p* *=* 0.026), and unsaturated iron binding concentration (UIBC; 425 ± 139 vs. 357 ± 100; *p* *=* 0.015) were significantly higher in the anemic group. RBC folate and vitamin B12 levels did not differ significantly between the two groups.

**Table 2:** Hematological markers in anemic vs. non-anemic patients.

Anemia Present				
Parameter	All Patients (*n* = 187)	Yes (*n* = 39)	No (*n* = 148)	*p*-value
Anemia Biomarkers				
Ferritin (μg/L)	28.8 ± 27.8	24.2 ± 33.3	30.0 ± 26.2	0.0021^b^^,^*
RBC (10^6^/uL)	4.12 ± 0.46	3.96 ± 0.56	4.16 ± 0.43	0.0048^b^^,^*
HCT (%)	36.3 ± 3.06	32.5 ± 2.3	37.3 ± 2.4	<0.001^a^^,^*
MCV (fL)	88.6 ± 7.5	83.2 ± 9.4	90.0 ± 6.3	<0.001^b^^,^*
MCH (pg)	28.9 ± 3.0	26.3 ± 3.6	29.6 ± 2.4	<0.001^b^^,^*
MCHC (g/dL)	32.6 ± 1.1	31.5 ± 1.2	32.9 ± 0.9	<0.001^a^^,^*
TIBC (μg/dL)	448 ± 98	487 ± 119	438 ± 90	0.026^b^^,^*
UIBC (μg/dL)	371 ± 112	425 ± 139	357 ± 100	0.015^b^^,^*
RBC Folate (ng/mL)	715 ± 324	726 ± 386	712 ± 308	0.82^a^
Folate (ng/mL)	14.3 ± 6.6	11.7 ± 6.6	14.9 ± 6.5	0.0058^a^^,^*
Vitamin B12 (pg/mL)	317 ± 152	274 ± 114	328 ± 159	0.14^b^

RBC, red blood cell; HCT, hematocrit; MCV, mean corpuscular volume; MCH, mean corpuscular hemoglobin; MCHC, mean corpuscular hemoglobin concentration; TIBC, total iron binding capacity; UIBC, unsaturated iron binding capacity; values presented as mean ± SD.

* *p*-value <0.05.

^a^ Student's *t*-test.

^b^ Wilcoxon rank-sum test.

On the day of recruitment, participants were asked to report the pregnancy symptoms they experienced from a predefined list of commonly experienced symptoms during pregnancy. The results are listed in Table 3, where the participants were stratified by the severity of anemia. Two participants from the non-anemic group were excluded from the analysis due to incomplete responses in the relevant questionnaire section. The proportion of individuals experiencing self-perceived anemic symptoms (such as tiredness, headache, dizziness) compared to those without symptoms was significantly different among the three groups (*p* *=* 0.023). A closer look at the proportion reveals that 10% and 20% of those with moderate or mild anemia had self-perceived anemic symptoms, compared to only 5.5% in healthy individuals. The proportion of individuals experiencing bloating (*p* *=* 0.063) and heartburn (*p* *=* 0.052) between the three groups had variations in trend but did not reach statistical significance. Other symptoms, such as constipation, nausea, edema, and muscle spasms, were insignificant between groups.

**Table 3:** Common pregnancy symptoms present in anemic vs. non-anemic individuals.

Anemic				
Symptoms	Non-Anemic^a^ (*n* = 146)	Mild (*n* = 29)	Moderate (*n* = 10)	*p*-value
Self-perceived Anemia				
Yes	8 (5.5)	6 (20.7)	1 (10.0)	0.023*
No	138 (94.5)	23 (79.3)	9 (90.0)	
Constipation				
Yes	49 (33.6)	12 (41.4)	3 (30.0)	0.69
No	97 (66.4)	17 (58.6)	7 (70.0)	
Bloating				
Yes	73 (50.0)	9 (31.0)	7 (70.0)	0.063
No	73 (50.0)	20 (69.0)	3 (30.0)	
Nausea				
Yes	74 (50.7)	17 (58.6)	6 (60.0)	0.65
No	72 (49.3)	12 (41.4)	4 (40.0)	
Heartburn				
Yes	8 (5.5)	5 (17.2)	0 (0.0)	0.052
No	138 (94.5)	24 (82.8)	10 (10.0)	
Edema				
Yes	20 (13.7)	4 (13.8)	2 (20.0)	0.86
No	126 (86.3)	25 (86.2)	8 (80.0)	
Muscle Spasm				
Yes	46 (31.5)	9 (31.0)	2 (20.0)	0.75
No	100 (68.5)	20 (69.0)	8 (80.0)	

Values presented as (%); values calculated by Chi-squared test.

* *p*-value < 0.05.

^a^ Some data removed because of unavailability.

## 4 Discussion

This study recruited 187 women with a mean age of 33.4 ± 4.2 years. Participants were classified as anemic if their hemoglobin levels were ≤10.9 mg/dL, and they were separated into two groups based on this classification. There was no significant difference in age between the two groups.

No correlation was found between education status and anemia in this study. This finding differs from studies conducted in Nepal by Yadav et al. and Sunuwar et al., as well as a study conducted in Iran by Khani Jeihooni et al. (11–13). The difference may be explained by the availability of national health insurance in Taiwan, which provides healthcare services and knowledge for pregnancy preparation and postnatal care (14). This could have mitigated the effect of inadequate formal education on anemia during pregnancy. Income was also not correlated with the prevalence of anemia, which is supported by a recent study indicating that high-income countries have an overall lower prevalence of anemia regardless of income level (15, 16). The difference in income levels may be less pronounced in these countries due to their higher standard of living and access to healthcare. The proportion of participants carrying female babies was higher in the group diagnosed with anemia. However, there is currently no evidence regarding the relationship between the infant's sex and anemia in pregnant individuals. Future studies should explore whether there is an association between the two variables.

The correlation between BMI and anemia remains inconsistent. A study by Eltayeb et al., which included 334 pregnant women, found no correlation between body weight status and anemia (17). This finding is consistent with our study and several other similar studies (17–19). When analyzing the relationship between pregnancy trimester and anemia, we found that the proportion of participants in the third trimester of pregnancy was significantly higher in the anemic group. This could be due to the increased iron requirement for fetal growth (20, 21), which may explain the lower Hb concentration observed in this group of individuals. Those in the first trimester of pregnancy are also more susceptible to lower Hb levels due to an increase in plasma volume (21). Anemia at any stage of pregnancy can have detrimental effects on both the mother and the baby, including intrauterine growth retardation, intrauterine death, preterm birth, low birth weight, increased risk of small-for-gestational-age babies, and increased neonatal intensive care unit admission (22). Additionally, participants with anemia during the first trimester of pregnancy demonstrate a higher prevalence of anxiety and depression postpartum (23). This highlights the importance of anemia prevention before and during pregnancy by ensuring adequate iron stores through proper dietary intake (24).

The hematological markers that were found to be significantly different in this study are widely recognized as markers altered in patients with anemia. Our findings regarding ferritin are consistent with current literature (25). Ferritin is associated with long-term iron storage. It is considered the most sensitive marker for iron deficiency when no inflammatory condition is present. When the supply of dietary iron is insufficient, ferritin levels decrease (26, 27). This explains why ferritin levels are significantly lower in patients with anemia, although they are still higher than the cut-off used for anemia in studies (27). The decreased levels of RBC (28), hematocrit (29), MCV, MCH, and MCHC in the anemia group are also consistent with the current findings (30).

In this study, plasma folate levels were significantly lower in individuals with anemia but still within the normal range. Although folate is not directly related to iron deficiency anemia, its role in preventing megaloblastic anemia and neural tube defects in infants has long been documented (31). Thus, biomarker tests are essential to differentiate between different subtypes of anemia and prevent adverse effects caused by nutrient deficiencies. When combined with MCV readings, the smaller volume of RBCs in the anemic group suggests that anemia is not solely attributable to low folate levels. Additionally, RBC folate, a more reliable long-term marker (32), did not differ significantly between the two groups. Conversely, TIBC and UIBC levels are elevated in individuals with anemia. TIBC refers to transferrin concentration in the blood, which increases with higher iron demand (33). Inadequate iron intake from dietary sources, coupled with increased iron demand during pregnancy, leads to increased synthesis of transferrin to prepare the body for greater uptake of exogenous iron. This explains the increase in UIBC also observed in a similar study of pregnant women with anemia (34).

Pregnancy can bring about various discomforts, with symptoms varying between individuals. A recent study by Nissen et al. found that each symptom may have a specific trimester of onset (35). Breathlessness, fatigue, and heartburn peak during the third trimester due to physiological needs and hormonal changes (35, 36), in which the prevalence of breathlessness and fatigue aligns with the findings of this study. Anemia is also often characterized by clinical manifestations such as breathlessness and fatigue (37). It can be inferred that the presence of symptoms of anemia may present itself similarly to pregnancy symptoms and should not be dismissed. The lack of significant differences in RBC folate and vitamin B12 levels between the two groups, combined with the normal MCV, suggests that these symptoms were likely not caused by deficiencies in these two vitamins (38).

Although the increased prevalence of bloating with anemia progression did not reach statistical significance (*p* *=* 0.063), further investigation is necessary to confirm this marginal significance. This is particularly important as anemic individuals may experience gastrointestinal symptoms that often go unnoticed (39). The established link between anemia and gastrointestinal problems (40, 41) highlights the need for further study in this area, particularly in the Taiwanese population. While the presence of heartburn approached near-significance (*p* *=* 0.052), we did not observe a distinct pattern between the groups. Heartburn was mainly present in pregnant individuals with mild anemia (17.2%) and was absent in those with moderate anemia. In pregnant individuals, heartburn can be attributed to fluctuating hormone levels that affect the lower esophageal sphincter and alter gastrointestinal motility (42). It should be underlined that the associations between bloating or heartburn and anemia in pregnant individuals require further study with larger sample cohorts to verify these observations.

In this study, we found that the presence of constipation, nausea, edema, and muscle spasms did not differ between the anemic and non-anemic groups. Constipation and nausea during pregnancy, as mentioned above, are rooted in physiological and hormonal changes, including human chorionic gonadotropin, estradiol, thyroid hormones (43), estrogen, and progesterone (44), among others. While the relationship between gastrointestinal symptoms and anemia in pregnant women has not been explored, iron supplementation has been implicated with constipation in non-pregnant individuals (45). Edema and muscle spasms during pregnancy also commonly occur, with higher prevalence and severity during the third trimester (46, 47). On the other hand, the mechanism of edema in chronic anemia is well understood (48). Anemia impairs normal muscle functioning due to the lack of oxygen supply, resulting in abnormal energy metabolism and muscle spasms (49). However, the similar prevalence of edema and muscle spasms between anemic and non-anemic individuals in this study is likely attributable to the relatively mild severity of anemia.

The strength of this study lies in its use of comprehensive blood test panels, which allowed for detailed analysis. By incorporating both subjective and objective data, the study enhances the utility of non-invasive testing in a clinical setting. However, several limitations must be noted. This exploratory study utilizes convenience sampling, which may cause sampling bias and limit generalizability to the population. Secondly, the cross-sectional nature of the study limits the ability to establish causal relationships, and some questions, including the presence of pregnancy symptoms, were reliant on participant perception and recall.

## 5 Conclusion

In conclusion, this study found that self-perceived anemia symptoms (such as tiredness, fatigue, and shortness of breath) were reported more frequently among pregnant individuals with clinical anemia. Aside from lower-than-normal Hb levels, pregnant individuals with anemia have lower ferritin, RBC count, HCT, MCV, MCH, MCHC, and plasma folate, and higher TIBC and UIBC when compared to healthy pregnant individuals. These observations imply that, aside from altered biomarkers, the presence of self-perceived anemia symptoms may provide an indication for the presence of anemia. While the symptoms of anemia and pregnancy may overlap, we suggest that when the symptoms present themselves, anemia screening should be done when possible to confirm the presence of clinical anemia. This may facilitate early detection and intervention to mitigate the negative effects of anemia on the mother and fetus.

## Data Availability

The original contributions presented in the study are included in the article/Supplementary Material, further inquiries can be directed to the corresponding author.
