# Supplementary material for: Common symptoms in pregnancy and their correlations with anemia
Source: Front Glob Womens Health. 2026 Oct 26;7:1554655. doi: 10.3389/fgwh.2026.1554655 (PMC13745659; doi:10.3389/fgwh.2026.1554655)
Supplement: Supplementary file 1 [file Table1.docx]

STROBE Statement—checklist of items that should be included in reports of observational studies

|  | | | Item No. | | Recommendation | | | Page  No. | | | | | | | | | | | | | | | Relevant text from manuscript | | | | | | |  |  |
| --- | --- | --- | --- | --- | --- | --- | --- | --- | --- | --- | --- | --- | --- | --- | --- | --- | --- | --- | --- | --- | --- | --- | --- | --- | --- | --- | --- | --- | --- | --- | --- |
| **Title and abstract** | | | 1 | | (*a*) Indicate the study’s design with a commonly used term in the title or the abstract | | | 1  4 | | | | | | | | | | | | | | | “This cross-sectional study analyzed data from 187 women who were recruited during their pregnancy, along with 37 of their infants.”  “This cohort study enrolled pregnant women from the Outpatient Department..” | | | | | | |  |  |
|  |  |  |  |  | (*b*) Provide in the abstract an informative and balanced summary of what was done and what was found | | | 1 | | | | | | | | | | | | | | | “This cross-sectional study analyzed data from 187 women who were recruited during their pregnancy, along with 37 of their infants. A higher percentage of those who reported symptoms of anemia, such as tiredness and dizziness, were found to have clinical anemia (20.7% in mildly anemic individuals and 10.0% in moderately anemic individuals, *p=*0.023). Bloating (31.0% in mildly anemic individuals, 70.0% in moderately anemic individuals) and heartburn (17.0% in mildly anemic individuals) were also more common in mothers who experienced anemia during pregnancy, although these differences did not reach statistical significance (*p=*0.65 and *p=*0.052, respectively).” | | | | | | |  |  |
| Introduction | | | | | | | | | | | | | | | | | | | | | | | |  | | |  |  |  |  |  |
| Background/rationale | | | 2 | | | Explain the scientific background and rationale for the investigation being reported | | | | 1 | | | | | | | | “Anemia remains a significant global health problem that affects people of all ages and sexes..” | | | | | | | | | |  |  |  |  |
| Objectives | | | 3 | | | State specific objectives, including any prespecified hypotheses | | | | 2 | | | | | | | | “Given the urgency of addressing this condition in the population, studying the correlation between anemia and pregnancy symptoms and infant biomarkers is crucial.” | | | | | | | | | |  |  |  |  |
| Methods | | | | | | | | | | | | | | | | | | | | | | | |  | | |  |  |  |  |  |
| Study design | | | 4 | | | Present key elements of study design early in the paper | | | | 2 | | | | | | “Biochemical values of mothers were obtained on the day of subject recruitment via a blood test as part of the routine checkup procedure during pregnancy. Outcome post-pregnancy was obtained from medical records during outpatient follow-up until childbirth. After giving birth, we asked the participants during their infant outpatient clinic follow-up to confirm if they still wished to participate in the infant study by signing a written informed consent. On the same day, breast milk samples were collected from the mothers. Plasma biochemical values of infants above 6 months of age were obtained through the infant's patient record. After analyzing the completeness of the information, 187 participants and 37 infants were eligible for inclusion in this study.” | | | | | | | | | | | | |  |  |  |
| Setting | | | 5 | | | Describe the setting, locations, and relevant dates, including periods of recruitment, exposure, follow-up, and data collection | | | | 2 | | | | | | “This cross-sectional study enrolled pregnant women from the Outpatient Department of Obstetrics and Gynecology, Taipei Tzu-Chi Hospital, New Taipei City, Taiwan (R.O.C.), between November 2018 and August 2019.”  “Biochemical values of mothers were obtained on the day of subject recruitment via a blood test as part of the routine checkup procedure during pregnancy. Outcome post-pregnancy was obtained from medical records during outpatient follow-up until childbirth. After giving birth, we asked the participants during their infant outpatient clinic follow-up to confirm if they still wished to participate in the infant study by signing a written informed consent. On the same day, breast milk samples were collected from the mothers.” | | | | | | | | | | | | |  |  |  |
| Participants | | | 6 | | | (*a*) *Cohort study*—Give the eligibility criteria, and the sources and methods of selection of participants. Describe methods of follow-up  *Case-control study*—Give the eligibility criteria, and the sources and methods of case ascertainment and control selection. Give the rationale for the choice of cases and controls  *Cross-sectional study*—Give the eligibility criteria, and the sources and methods of selection of participants | | | | 2 | | | | | | “This cross-sectional study enrolled pregnant women from the Outpatient Department of Obstetrics and Gynecology, Taipei Tzu-Chi Hospital, New Taipei City, Taiwan (R.O.C.), between November 2018 and August 2019. The participants were recruited through convenient sampling. The inclusion criteria were as follows: (1) Pregnant women aged 15–45 with no major comorbidities (e.g. cancer, diabetes, cardiovascular disease); (2) complete anthropometric and biochemical data.”  “Biochemical values of mothers were obtained on the day of subject recruitment via a blood test as part of the routine checkup procedure during pregnancy. Outcome post-pregnancy was obtained from medical records during outpatient follow-up until childbirth. After giving birth, we asked the participants during their infant outpatient clinic follow-up to confirm if they still wished to participate in the infant study by signing a written informed consent. On the same day, breast milk samples were collected from the mothers. Plasma biochemical values of infants above 6 months of age were obtained through the infant's patient record.” | | | | | | | | | | | | |  |  |  |
|  |  |  |  |  |  | (*b*) *Cohort study*—For matched studies, give matching criteria and number of exposed and unexposed  *Case-control study*—For matched studies, give matching criteria and the number of controls per case | | | | - | | | | | | - | | | | | | | | | | | | |  |  |  |
| Variables | | | 7 | | | Clearly define all outcomes, exposures, predictors, potential confounders, and effect modifiers. Give diagnostic criteria, if applicable | | | | 3 | | | | | | “The diagnosis of iron-deficiency anemia and the severity of anemia were grouped according to the serum hemoglobin (Hb) concentration using the criteria defined by the World Health Organization in the Vitamin and Mineral Nutrition Information System. A plasma Hb level of more than 10.9 g/dL during any pregnancy trimester was classified as adequate; 10.0 g/dL ≤ Hb ≤10.9 g/dL was classified as mild anemia; 7.0 g/dL ≤ Hb ≤9.9 g/dL was classified as moderate anemia; Hb <7.0 g/dL was classified as severe anemia. According to this classification, no individual was classified as having severe anemia. Infant anemia was classified using the cutoff point for infants and children. Body mass index (BMI) grouping was performed according to the adult BMI criteria defined by Taiwan’s Ministry of Health and Welfare as follows: (1) BMI <18.5 kg/m^2^ as underweight; (2) 18.5 kg/m^2^ ≤ BMI <24 kg/m^2^ as normal body weight; (3) 24 kg/m^2^ ≤ BMI <27 kg/m^2^ as overweight; (4) BMI ≥27 kg/m^2^ as obese.” | | | | | | | | | | | | |  |  |  |
| Data sources/ measurement | | | 8* | | | For each variable of interest, give sources of data and details of methods of assessment (measurement). Describe comparability of assessment methods if there is more than one group | | | | 3 | | | | | | “The diagnosis of iron-deficiency anemia and the severity of anemia were grouped according to the serum hemoglobin (Hb) concentration using the criteria defined by the World Health Organization in the Vitamin and Mineral Nutrition Information System. A plasma Hb level of more than 10.9 g/dL during any pregnancy trimester was classified as adequate; 10.0 g/dL ≤ Hb ≤10.9 g/dL was classified as mild anemia; 7.0 g/dL ≤ Hb ≤9.9 g/dL was classified as moderate anemia; Hb <7.0 g/dL was classified as severe anemia. According to this classification, no individual was classified as having severe anemia.  Infant anemia was classified using the cutoff point for infants and children. Body mass index (BMI) grouping was performed according to the adult BMI criteria defined by Taiwan’s Ministry of Health and Welfare as follows: (1) BMI <18.5 kg/m^2^ as underweight; (2) 18.5 kg/m^2^ ≤ BMI <24 kg/m^2^ as normal body weight; (3) 24 kg/m^2^ ≤ BMI <27 kg/m^2^ as overweight; (4) BMI ≥27 kg/m^2^ as obese.” | | | | | | | | | | | | |  |  |  |
| Bias | | | 9 | | | Describe any efforts to address potential sources of bias | | | | 3 | | | | | | “According to the variance and distribution of the data, the appropriate statistical analysis method was applied”  “A baseline comparison between the subpopulation with infant data and total participants has been done with no statistically significant difference.” | | | | | | | | | | | | |  |  |  |
| Study size | | | 10 | | | Explain how the study size was arrived at | | | | 3 | | | | | | “A total of 191 pregnant women were recruited for this study through convenient sampling.” | | | | | | | | | | | | |  |  |  |
| Quantitative variables | | | 11 | | | Explain how quantitative variables were handled in the analyses. If applicable, describe which groupings were chosen and why | | | | 3 | | | | | | “Results of categorical variables were presented as *n (%)*, with *n* representing the number of people in a certain group. The results of continuous variables were presented as mean ± SD.” | | | | | | | | | | | | |  |  |  |
| Statistical methods | | | 12 | | | (*a*) Describe all statistical methods, including those used to control for confounding | | | | 3 | | | | | | “According to the variance and distribution of the data, the appropriate statistical analysis method was applied, including the Student’s *t*-test, Wilcoxon rank-sum test, or chi-square test. The correlation coefficient was calculated using Pearson's or Kendall's rank correlation tests whenever appropriate.”  “Subgrouping according to different criteria (including pregnancy trimester, formal education, and income, among others) was done to control confounders. Missing data were removed from the analysis. A baseline comparison between the subpopulation with infant data and total participants has been done with no statistically significant difference.” | | | | | | | | | | | | |  |  |  |
|  |  |  |  |  |  | (*b*) Describe any methods used to examine subgroups and interactions | | | | 3 | | | | | | “According to the variance and distribution of the data, the appropriate statistical analysis method was applied, including the Student’s *t*-test, Wilcoxon rank-sum test, or chi-square test. The correlation coefficient was calculated using Pearson's or Kendall's rank correlation tests whenever appropriate. Results of categorical variables were presented as *n (%)*, with *n* representing the number of people in a certain group. The results of continuous variables were presented as mean ± SD. Correlation strength was classified as follows: 0.2–0.39 as weak, 0.40–0.59 as moderate, 0.6–0.79 as strong, and 0.8–1 as very strong.” | | | | | | | | | | | | |  |  |  |
|  |  |  |  |  |  | (*c*) Explain how missing data were addressed | | | | 3 | | | | | | “Missing data were removed from the analysis.” | | | | | | | | | | | | |  |  |  |
|  |  |  |  |  |  | (*d*) *Cohort study*—If applicable, explain how loss to follow-up was addressed  *Case-control study*—If applicable, explain how matching of cases and controls was addressed  *Cross-sectional study*—If applicable, describe analytical methods taking account of sampling strategy | | | | 2 | | | | | | “The participants were recruited through convenient sampling.” | | | | | | | | | | | | |  |  |  |
|  |  |  |  |  |  | (*e*) Describe any sensitivity analyses | | | | 3 | | | | | | “Subgrouping according to different criteria (including pregnancy trimester, formal education, and income, among others) was done to control confounders.” | | | | | | | | | | | | |  |  |  |
| Results | | | | | | | | | | | | | | | | | | | | | | | | |  |  |  |  |  |  |  |
| Participants | 13* | | | (a) Report numbers of individuals at each stage of study—eg numbers potentially eligible, examined for eligibility, confirmed eligible, included in the study, completing follow-up, and analysed | | | | | | | | | | 3 | | | | | | | | | “A total of 191 pregnant women were recruited in this study. After removing participants with incomplete data, 187 pregnant women were included in the study, with 39 women (20.9%) diagnosed with anemia and 148 women defined as non-anemic based on serum Hb levels” | | | | | | | | |
|  |  |  |  | (b) Give reasons for non-participation at each stage | | | | | | | | | | - | | | | | | | | | - | | | | | | | | |
|  |  |  |  | (c) Consider use of a flow diagram | | | | | | | | | | - | | | | | | | | | - | | | | | | | | |
| Descriptive data | 14* | | | (a) Give characteristics of study participants (eg demographic, clinical, social) and information on exposures and potential confounders | | | | | | | | 3 and Table I | | | | | | | | | | | “The baseline data of the participants collected on the day of recruitment are shown in Table I.” | | | | | | | | |
|  |  |  |  | (b) Indicate number of participants with missing data for each variable of interest | | | | | | | | Table I, Table III, Table IV | | | | | | | | | | | Shown in each table | | | | | | | | |
|  |  |  |  | (c) *Cohort study*—Summarise follow-up time (eg, average and total amount) | | | | | | | | - | | | | | | | - | | | | | | | | | | | | |
| Outcome data | 15* | | | *Cohort study*—Report numbers of outcome events or summary measures over time | | | | | | | | - | | | | | | | - | | | | | | | | | | | | |
|  |  |  |  | *Case-control study—*Report numbers in each exposure category, or summary measures of exposure | | | | | | | | - | | | | | | | - | | | | | | | | | | | | |
|  |  |  |  | *Cross-sectional study—*Report numbers of outcome events or summary measures | | | | | | | | 6-8,  Table I-V | | | | | | | Shown in Results and each table | | | | | | | | | | | | |
| Main results | 16 | | | (*a*) Give unadjusted estimates and, if applicable, confounder-adjusted estimates and their precision (eg, 95% confidence interval). Make clear which confounders were adjusted for and why they were included | | | | | 3-4,  Table I-V  5-6 | | | | | | | | | | Shown in Results and each table  Reasons for subgrouping available in Discussion | | | | | | | | | | | | |
|  |  |  |  | (*b*) Report category boundaries when continuous variables were categorized | | | | | | | | | 3 | | | | | | | | “A plasma Hb level of more than 10.9 g/dL during any pregnancy trimester was classified as adequate; 10.0 g/dL ≤ Hb ≤10.9 g/dL was classified as mild anemia; 7.0 g/dL ≤ Hb ≤9.9 g/dL was classified as moderate anemia; Hb <7.0 g/dL was classified as severe anemia.”  “Body mass index (BMI) grouping was performed according to the adult BMI criteria defined by Taiwan’s Ministry of Health and Welfare as follows: (1) BMI <18.5 kg/m^2^ as underweight; (2) 18.5 kg/m^2^ ≤ BMI <24 kg/m^2^ as normal body weight; (3) 24 kg/m^2^ ≤ BMI <27 kg/m^2^ as overweight; (4) BMI ≥27 kg/m^2^ as obese.” | | | | | | | | | |  |
|  |  | | | (*c*) If relevant, consider translating estimates of relative risk into absolute risk for a meaningful time period | | | | | | | | | - | | | | | | | | - | | | | | | | | | |  |
| Other analyses | 17 | | | Report other analyses done—eg analyses of subgroups and interactions, and sensitivity analyses | | | 3-4,  Table I-V | | | | | | | | | | | | | | | Shown in Results and each table | | | | | | | | |  |
| Discussion | | | | | | | | | | | | | | |  |  |  |  |  |  |  |  |  |  |  |  |  |  |  |  |  |
| Key results | 18 | | | Summarise key results with reference to study objectives | | | | | | | 7 | | | | | | | | | “In conclusion, this study found correlations between self-perceived anemia symptoms (such as tiredness, fatigue, and shortness of breath), and possibly some common pregnancy symptoms such as bloating and heartburn, and the presence of anemia. Although these symptoms may be present in healthy individuals due to hormonal and physiological changes, they are more common in anemic pregnant individuals.” | | | | | | | | | | |  |
| Limitations | 19 | | | Discuss limitations of the study, taking into account sources of potential bias or imprecision. Discuss both direction and magnitude of any potential bias | | | | | | | 7 | | | | | | | | | “The strength of this study lies in its use of comprehensive blood test panels, which allowed for detailed analysis. By incorporating both subjective and objective data, the study enhances the utility of non-invasive testing in a clinical setting. However, several limitations must be noted. For instance, information pertaining to the mother's hematological markers was only collected on the day of recruitment, which precludes establishing causation. Additionally, the small sample size of infants analyzed in subsequent evaluations limits the generalizability of the findings to the broader population.” | | | | | | | | | | |  |
| Interpretation | 20 | | | Give a cautious overall interpretation of results considering objectives, limitations, multiplicity of analyses, results from similar studies, and other relevant evidence | | | | | | | 7 | | | | | | | | | “In conclusion, this study found correlations between self-perceived anemia symptoms (such as tiredness, fatigue, and shortness of breath), and possibly some common pregnancy symptoms such as bloating and heartburn, and the presence of anemia. Although these symptoms may be present in healthy individuals due to hormonal and physiological changes, they are more common in anemic pregnant individuals. Anemia during gestation is associated with a higher prevalence of anemia in infants over 6 months after birth, increased infant transferrin levels, and decreased infant ferritin concentration.” | | | | | | | | | | |  |
| Generalisability | 21 | | | Discuss the generalisability (external validity) of the study results | | | | | | | 7 | | | | | | | | | “Additionally, the small sample size of infants analyzed in subsequent evaluations limits the generalizability of the findings to the broader population.” | | | | | | | | | | |  |
| Other information | |  | | | | | | | | | | | | | | | | | | | | | | | |  |  |  |  |  |  |
| Funding | 22 | Give the source of funding and the role of the funders for the present study and, if applicable, for the original study on which the present article is based | | | | | | | | | 3 | | | | | | “…and funding was provided under grant number TCRD-TPE-112-RT-9” | | | | | | | | | | | | | |  |
